# Molecular characterization of a flavanone 3-hydroxylase gene from citrus fruit reveals its crucial roles in anthocyanin accumulation

**DOI:** 10.1186/s12870-023-04173-3

**Published:** 2023-05-03

**Authors:** Gang Ma, Lancui Zhang, Risa Yamamoto, Nami Kojima, Masaki Yahata, Masaya Kato

**Affiliations:** 1 Department of Bioresource Sciences, Faculty of Agriculture, Shizuoka University, 836 Ohya, Suruga, Shizuoka 422-8529 Japan; 2 Graduate School of Integrated Science and Technology, Shizuoka University, 836 Ohya, Suruga, Shizuoka 422-8529 Japan

**Keywords:** Anthocyanin, Blue light, Citrus, Flavanone 3-hydroxylase, Juice sacs

## Abstract

**Background:**

Flavanone 3-hydroxylase (F3H), a key enzyme in the flavonoid biosynthetic pathway, plays an important role in the regulation of flavonols and anthocyanidins accumulation. Citrus fruit is a rich source of flavonoids with varied flavonoid compositions among different varieties. To date, the study on *F3H* is limited in citrus, and its roles in regulating flavonoid accumulation in citrus fruit are still unclear.

**Results:**

In this study, we isolated a *CitF3H* from three different citrus varieties, Satsuma mandarin (*Citrus unshiu* Marc.), Ponkan mandarin (*C*. *reticulata* Blanco) and blood orange ‘Moro’ (*C*. *sinensis* Osbeck). Functional analysis showed that *CitF3H* encoded a functional flavanone 3-hydroxylase. It catalyzed the hydroxylation of naringenin to yield dihydrokaempferol, which was a precursor of anthocyanins in flavonoid biosynthetic pathway. In the juice sacs, *CitF3H* was differentially expressed among the three citrus varieties, and its expression level was positively correlated with the accumulation of anthocyanins during the ripening process. In the juice sacs of Satsuma mandarin and Ponkan mandarin the expression of *CitF3H* kept constant at an extremely low level, and no anthocyanin was accumulated during the ripening process. In contrast, the expression of *CitF3H* increased rapidly along with the accumulation of anthocyanin in the juice sacs of blood orange ‘Moro’ during the ripening process. In addition, we found that blue light irradiation was effective to up-regulate the expression of *CitF3H* and improve anthocyanin accumulation in the juice sacs of blood orange ‘Moro’ in vitro.

**Conclusion:**

*CitF3H* was a key gene regulating anthocyanin accumulation in the juice sacs of citrus fruit. The results presented in this study will contribute to elucidating anthocyanin biosynthesis in citrus fruit, and provide new strategies to improve the nutritional and commercial values of citrus fruit.

**Supplementary Information:**

The online version contains supplementary material available at 10.1186/s12870-023-04173-3.

## Background

Flavonoids are a group of polyphenolic compounds with diverse structures. In nature, more than 8,000 different types of flavonoids have been identified, which are widely distributed in fruit, vegetables, and cereals. Flavonoids fulfill a variety of important functions in plants. They protect plants from various biotic and abiotic stresses, and act as signal molecules, allopathic compounds, detoxifying agents, and antimicrobial defensive compounds [1–4]. In addition, flavonoids are well known for their pharmacological properties. Flavonoids are one of the important compositions of herbal drugs, and have long been used in traditional medicine with high antioxidative, anti-inflammatory, anti-mutagenic and anti-carcinogenic activities [5–10]. Due to the indispensable benefits in plants and human health, flavonoids drew more and more attention of researchers over the past decades, and considerable progress has been made in the elucidation of flavonoid biosynthesis in plants [11–14].

Flavanone 3-hydroxylase (F3H), a member of 2-oxoglutarate-dependent dioxygenase family, is a key enzyme in the flavonoid biosynthetic pathway. Li et al. (2020) reported that F3Hs of seed plant (i.e., gymnosperms and angiosperms) were evolutionarily connected to flavone synthase Is (FNS Is) of liverworts [15]. The early-evolved FNS I has gone through functional transition as FNS I/F2H and FNS I/F3H, and eventually shifted to the bona fide F3H. The F3H gene was first reported in the flowers of *Antirrhinum majus*, and the homologous genes were subsequently isolated in other plant species, such as Arabidopsis, maize, torenia, *Reaumuria trigyna*, tea, wolfberry, and soybean [16–22]. In plants, F3H catalyzes the 3-hydroxylation of flavanones to form dihydroflavonols, which are precursors of flavonols and anthocyanins. *F3H* is the key gene in the regulation of flavonoid accumulation at the bifurcation of the flavonol and anthocyanin branches [16–18, 20]. Overexpression of *Lycium chinense F3H* and tea *F3H* significantly enhanced the contents of flavanols in tobacco [20, 21]. In strawberry, the RNAi-mediated silencing of *F3H* led to reduced anthocyanin levels, producing colorless torenia flower and strawberry fruit [23]. In addition, *F3H* not only regulates the flavonoid composition, but also plays an important role in stress resistance in plants. Under the abiotic stresses, the expression of *F3H* was induced along with the increases of flavonoids contents, which contributed to protecting the plants from oxidative damage [21, 24–27].

Citrus is a rich source of flavonoids, which are accumulated in the root, leaf, seed, and fruit. Citrus flavonoids are primarily classified into four groups: flavanones, flavones, flavonols, and anthocyanins according to the structure. The flavonoid composition varied greatly among different tissues and citrus varieties [28–30]. In citrus fruit, flavanones are accumulated as the major flavonoid, followed by flavones. In contrast, flavonols and anthocyanins are rarely accumulated in citrus fruit. In citrus, anthocyanins are specifically accumulated in blood oranges, such as ‘Moro’ and ‘Tarocco’, while most common citrus varieties are devoid of the ability to produce anthocyanins during the evolution [31, 32].

To date, the study on citrus *F3H* is limited, and its effects on the flavonoid accumulation in citrus fruit are still unclear [33–35]. In the present study, we isolated a *F3H* gene (*CitF3H*) from the two common citrus varieties, Satsuma mandarin and Ponkan mandarin, and one variety of blood orange, ‘Moro’. To elucidate the roles of *F3H* in citrus fruit, the function and expression of *CitF3H* were investigated in the juice sacs of three citrus varieties, and its regulation in response to blue and red light was characterized in vitro. The molecular characterization of *CitF3H* presented in this study contributed to elucidating flavonoid accumulation in citrus fruit.

## Results

### Isolation and sequence analysis of ***CitF3H***

**Fig. 1:**
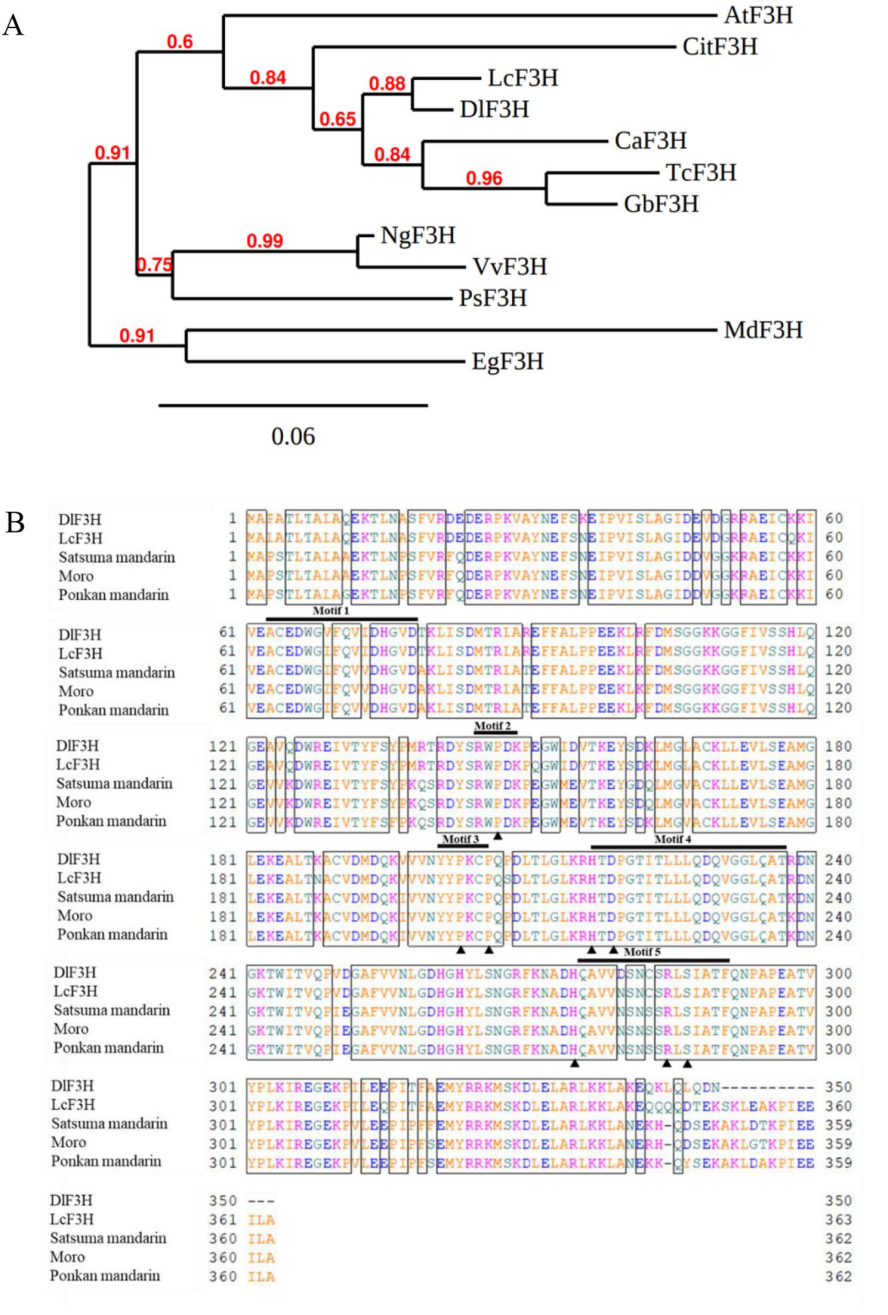
Phylogenetic analysis **(A)** and multiple sequence alignment **(B)** of CitF3H with other plant F3Hs. The amino acid sequences of *Citrus clementina* (CitF3H, Ciclev10025931m.g), Arabidopsis (AtF3H, AAC49176.1), *Litchi chinensis* (LcF3H, ADO95201.1), *Dimocarpus longan* (DlF3H, ABO48521.1), *Canarium album* (CaF3H, AEO36935.1), *Theobroma cacao* (TcF3H, XP_007046698.1), *Gossypium barbadense* (GbF3H, KAB2051536.1), *Nekemias grossedentata* (NgF3H, AFN70721.1), *Vitis vinifera* (VvF3H, NP_001268034.1), *Paeonia suffruticosa* (PsF3H, AEN71544.1), *Malus domestica* (MdF3H, NP_001280854.1), *Eustoma grandiflorum* (EgF3H, BAD34459.1) Satsuma mandarin (OQ148589), Ponkan mandarin (OQ148590), and blood orange ‘Moro’ (OQ148591) were used for phylogenetic tree analysis and multiple sequence analysis. The amino acid residues that were responsible for polypeptide folding, ferrous iron ligating, and 2-oxoglutarate binding were shown in the rectangle

In this study, to isolate the citrus flavanone 3-hydroxylase gene (*CitF3H*), a blast search in the *Citrus clementina* v.10 genome databases (http://www.phytozome.net/) was performed using the sequence of *LcF3H* as a query, which has been reported to encode a functional flavanone 3-hydroxylase in *Lycium chinese*. In citrus, *CitF3H* gene (Ciclev10025931m.g) was identified in the *Citrus clementina* v.10 genome databases, which was located at scaffold_7:1207140.1210820 (forward). The gene structure of *CitF3H* included a 5’ untranslated region, a coding sequence, and a 3’ untranslated region. In this study, *CitF3H* was isolated from three citrus varieties, Satsuma mandarin (Accession number: OQ148589), Ponkan mandarin (Accession number: OQ148590), and blood orange ‘Moro’ (Accession number: OQ148591). The nucleotide sequence of *CitF3H* contained 1,089 bp and encoded a putative protein of 362 amino acids with an estimated molecular mass of 40 kD. In the N-terminal region of the protein encoded by *CitF3H*, no characteristic transit peptide was predicted by TargetP-2.0 (http://www.cbs.dtu.dk/services/TargetP/). The deduced amino acid sequence of *CitF3H* showed more than 80% homology with *F3Hs* reported in other plant species (Table S1). A phylogenetic analysis showed that CitF3H was clustered with F3Hs from *Canarium album* (CaF3H), *Litchi chinensis* (LcF3H), *Gossypium barbadense* (GbF3H), *Theobroma cacao* (TcF3H), and *Dimocarpus longan* (DlF3H) (Fig. 1A). In addition, the amino acid sequence of *CitF3H* shared high identities (more than 97%) among the three varieties, Satsuma mandarin, Ponkan mandarin, and blood orange ‘Moro’ (Fig. 1B). Alignment of amino acid sequences of CitF3H with other plant F3H proteins showed that CitF3H contained five similar motifs for 2-oxoglutarate-dependent dioxygenase. Three prolines (Pro147, Pro203, and Pro206), which were responsible for polypeptide folding, were strictly conserved in the CitF3H of the three citrus varieties. Moreover, the amino acid residues (His217, Asp219, and His275) that were responsible for ferrous iron ligating, and the amino acid residues (Arg285 and Ser287) that were involved in the 2-oxoglutarate binding were also detected in the CitF3H of the three citrus varieties (Fig. 1B). The conserved motifs and amino acids residues detected in CitF3H suggested that CitF3H has potential functions in the biosynthesis of flavonoids in citrus fruit.

### Function analysis of recombinant ***CitF3H*** protein in vitro

**Fig. 2:**
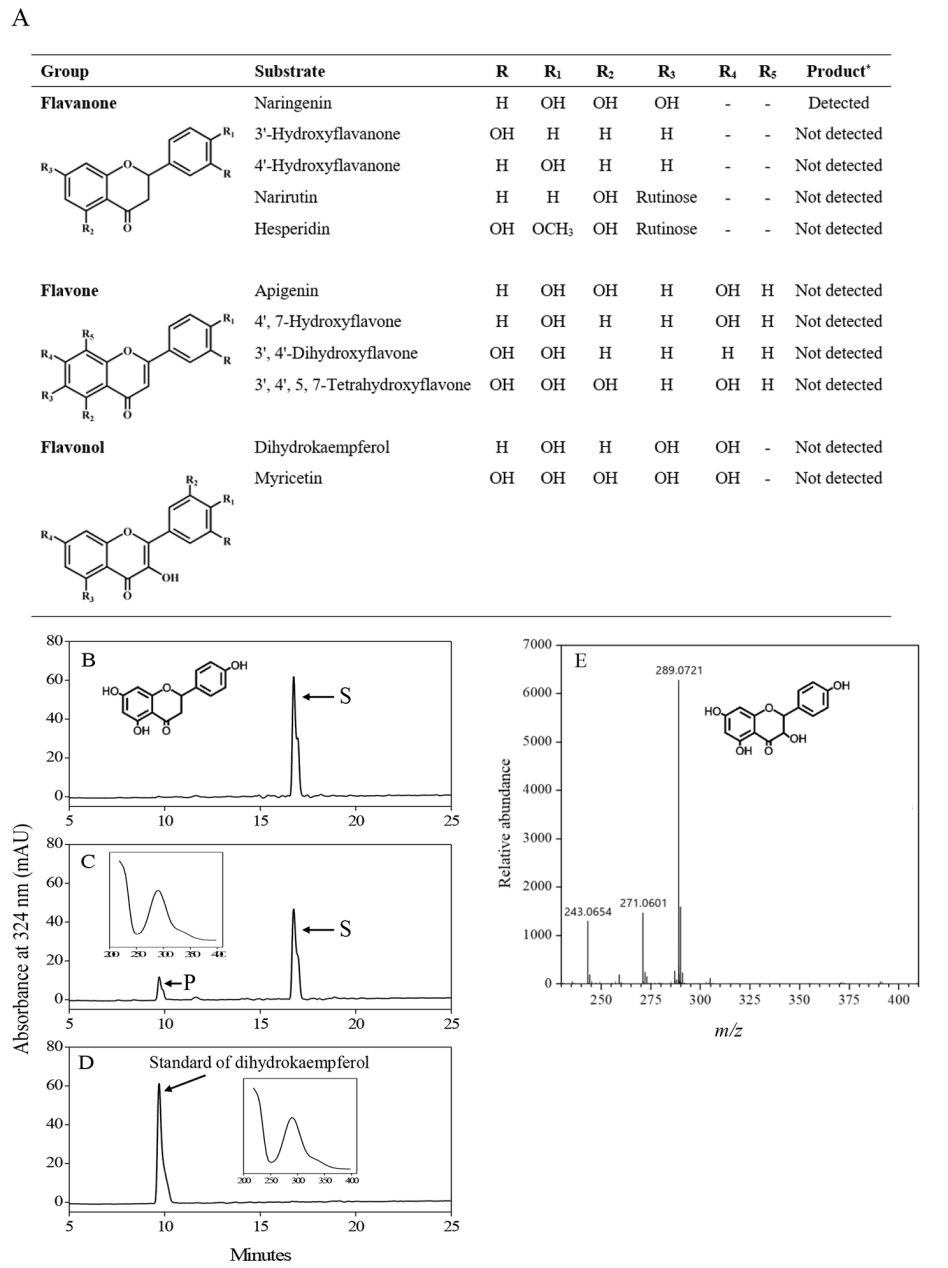
The enzyme activity of CitF3H in vitro. **(A)** Activities of CitF3H against different flavonoid substrates. **(B)** HPLC analysis of naringenin. **(C)** HPLC analysis of new hydroxylated product of naringenin catalyzed by CitF3H. **(D)** HPLC analysis of dihydrokaempferol standard. **(E)** Mass spectrum of the new hydroxylated product of naringenin catalyzed by CitF3H. S: naringenin, P: naringenin reaction product. * “Detected” or “Not detected” indicates whether there was a new peak detected by HPLC analysis

To investigate the function of *CitF3H*, the full-length cDNA of *CitF3H* isolated from Ponkan mandarin was cloned into the pCold GST vector. The recombinant protein expressed in *E.coli* cells was extracted and a single band with about 40 kDa molecular mass was confirmed by SDS-PAGE, which was in agreement with the prediction. In this study, to assay the enzyme activity of CitF3H, five flavanones (naringenin, 3’-hydroxyflavanone, 4’-hydroxyflavanone, narirutin, and hesperidin), four flavones (apigenin, 4’,7-hydroxyflavone, 3’,4’-dihydroxyflavone, and 3’,4’,5,7-tetrahydroxyflavone), and two flavanols (dihydrokaempferol and myricetin) were used as substrates. As shown in Fig. 2A, when naringenin was used as a substrate, a new product was detected. Whereas, no new product was observed when other flavonoids were used as substrates. In the case of naringenin, the new product was eluted at 9.7 min; the elution time and absorption maximum of the new product were identical to the standard of dihydrokaempferol (Fig. 2B–E). In addition, we also investigated the function of CitF3H by using in vivo feeding experiments. The *E.coli* cells harboring the pGEX-6P-1-CitF3H construct were fed with naringenin, and HPLC analysis of the reaction product confirmed that dihydrokaempferol was produced as in vitro reactions (Fig. S1). These results suggested that *CitF3H* encoded a functional flavanone 3-hydroxylase, and it catalyzed the formation of dihydrokaempferol from naringenin in flavonoid biosynthetic pathway.

### Changes in the expression of ***CitF3H*** and flavonoids contents in the juice sacs of Satsuma mandarin, Ponkan mandarin, and blood orange ‘Moro’ during the ripening process

**Fig. 3:**
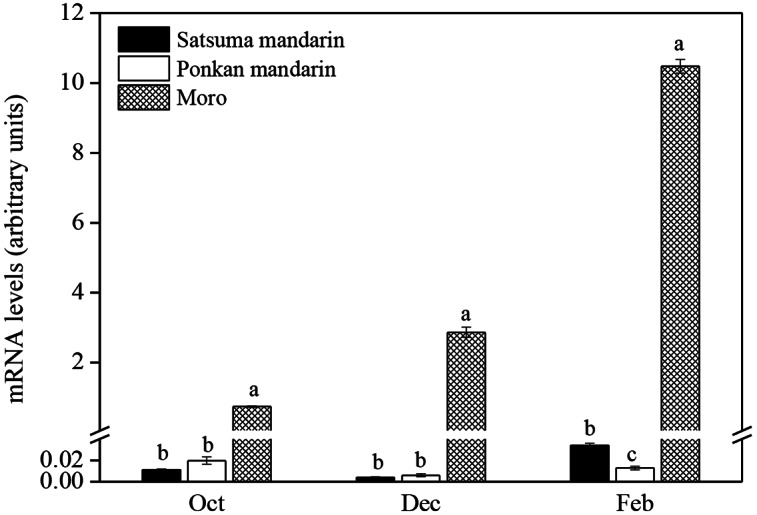
Gene expression analysis of *CitF3H* in the juice sacs of Satsuma mandarin, Ponkan mandarin, and blood orange ‘Moro’ during the ripening process. The mRNA levels were analyzed by TaqMan real-time quantitative PCR. 18 S ribosomal RNA was used to normalize the expression of gene in the same conditions. Columns and bars represent the means ± SE (n = 3), respectively. Tukey’s HSD test (*P* < 0.05) was used to compare the different varieties. Different letters above each column indicate significant differences of the gene expression of *CitF3H* among the three citrus varieties in October, December, and February, respectively. Oct: October, Dec: December, Feb: February

As shown in Fig. 3, *CitF3H* was differentially expressed in the juice sacs among Satsuma mandarin, Ponkan mandarin, and blood orange ‘Moro’. During the ripening process, the expression of *CitF3H* kept constant at an extremely low level in the juice sacs of Satsuma mandarin and Ponkan mandarin (Fig. 3). In blood orange ‘Moro’, in contrast, the expression of *CitF3H* increased rapidly in the juice sacs during the ripening process. The expression level of *CitF3H* in the juice sacs of blood orange ‘Moro’ was much higher than that in Satsuma mandarin and Ponkan mandarin during the ripening process.

**Fig. 4:**
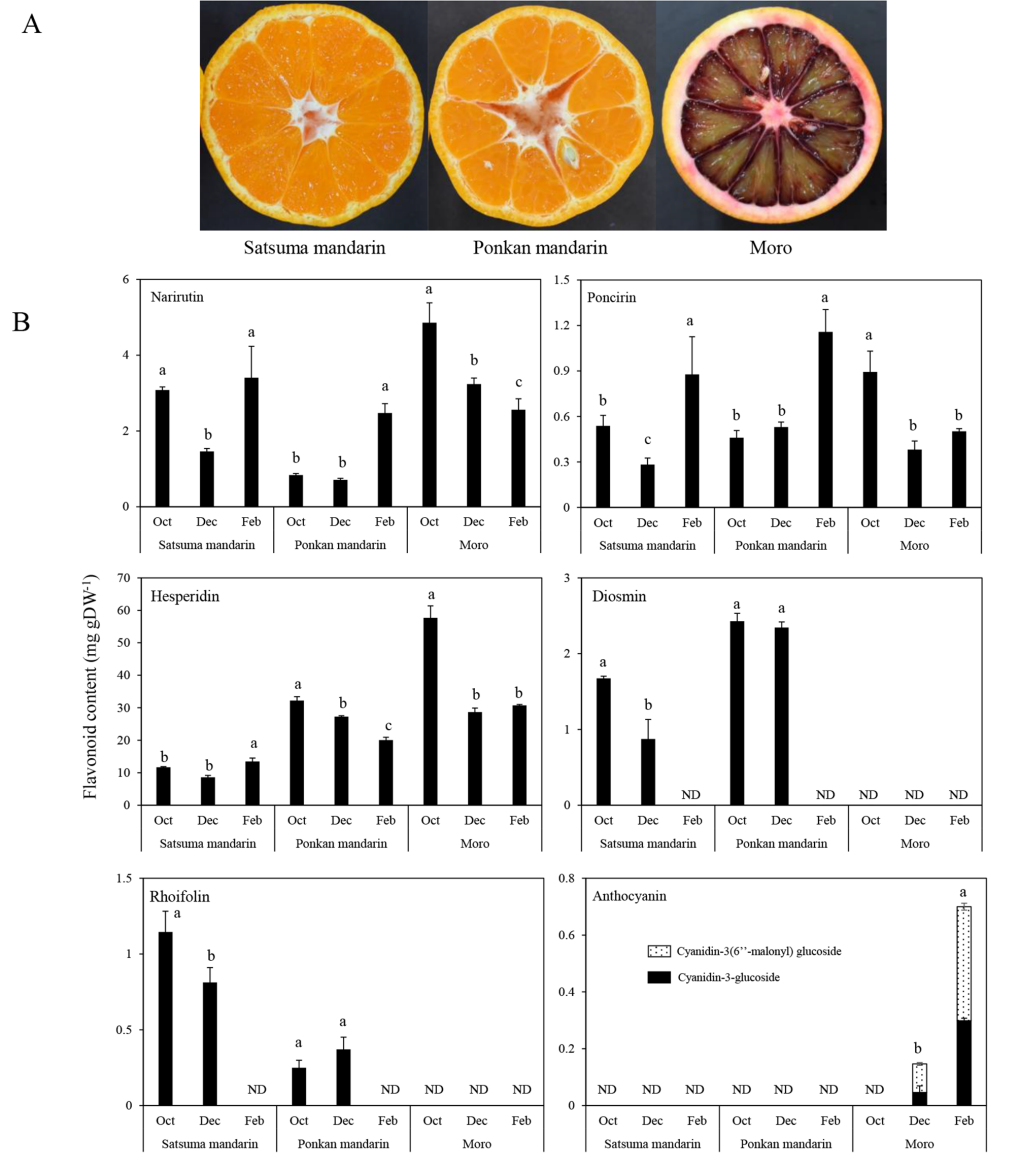
Flavonoid accumulation in the juice sacs of Satsuma mandarin, Ponkan mandarin, and blood orange ‘Moro’ during the ripening process. **(A)** The appearance of the juice sacs of Satsuma mandarin, Ponkan mandarin, and blood orange ‘Moro’; **(B)** Flavonoid content in the juice sacs of Satsuma mandarin, Ponkan mandarin, and blood orange ‘Moro’. Columns and bars represent the means ± SE (n = 3), respectively. Different letters above each column indicate significant differences at *P* < 0.05 by Tukey’s HSD test. ND, not detected. Oct: October, Dec: December, Feb: February

In this study, to elucidate the roles of *CitF3H* in the regulation of flavonoid accumulation in citrus fruit, the changes in the flavonoid content and composition were investigated in the juice sacs of the three citrus varieties during the ripening process. In Satsuma mandarin and Ponkan mandarin, three flavanones (narirutin, poncirin, and hesperidin) and two flavones (diosmin and rhoifolin) were accumulated in the juice sacs (Fig. 4). In blood orange ‘Moro’, the three flavanones were accumulated in the juice sacs, while flavones were not detected during the ripening process. In this study, we did not detect the flavonols in the juice sacs of Satsuma mandarin, Ponkan mandarin, or blood orange ‘Moro’ during the ripening process.

In addition, we found that a major difference in the flavonoid composition among the three citrus varieties was the accumulation of anthocyanins. In blood orange ‘Moro’, two anthocyanins, cyanidin-3-glucoside and cyanidin-3(6’’-malonyl) glucoside, were accumulated in the juice sacs (Fig. 4). During the ripening process, the contents of cyanidin-3-glucoside and cyanidin-3(6’’-malonyl) glucoside increased rapidly in the juice sacs of blood orange ‘Moro’, which was well consistent with the increase in the expression of *CitF3H* during the ripening process. In contrast, anthocyanins were undetectable in the juice sacs of Satsuma mandarin and Ponkan mandarin during the ripening process.

### Changes in the expression of ***CitF3H*** and flavonoid content in the juice sacs of blood orange ‘Moro’ in response to blue and red LED lights irradiation

**Fig. 5:**
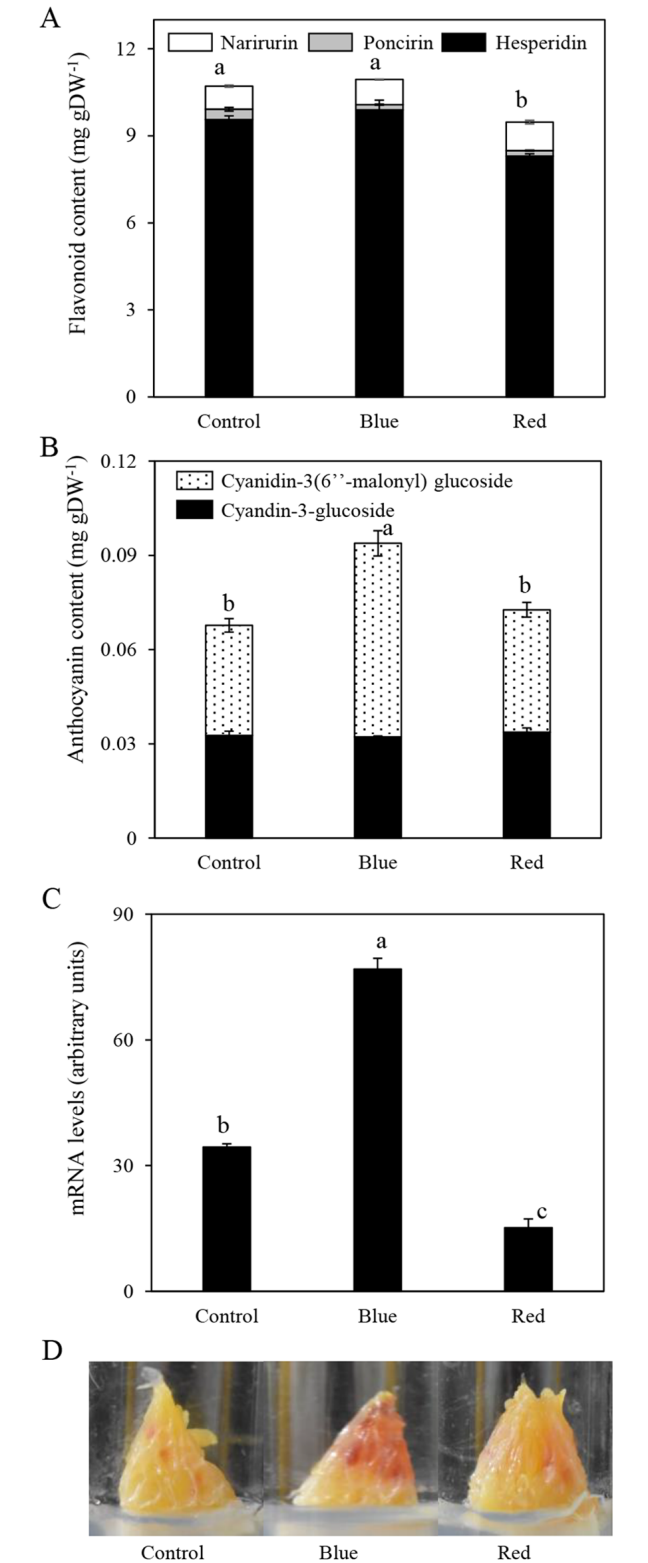
Effect of blue and red LED lights on flavonoid content **(A)**, anthocyanin content **(B)**, gene expression of *CitF3H* **(C)**, and appearance of the juice sacs in blood orange ‘Moro’ in vitro **(D)**. The mRNA levels were analyzed by TaqMan real-time quantitative PCR. 18 S ribosomal RNA was used to normalize the expression of gene in the same conditions. Columns and bars represent the means ± SE (n = 3), respectively. Different letters above each column indicate significant differences at *P* < 0.05 by Tukey’s HSD test

In this study, the effects of blue and red LED lights on the flavonoid content and the expression of *CitF3H* were investigated in the juice sacs of blood orange ‘Moro’ in vitro. Under the blue light, the contents of narirutin, poncirin, and hesperidin were not significantly affected, and the total flavonoid content in the blue light treatment was similar to that of the control (Fig. 5A). Under the red light, the content of hesperidin was decreased, and as a result the total flavonoid content in the red-light treatment was lower than that of the control (Fig. 5A). In addition, we found that the blue light treatment induced anthocyanin accumulation in the juice sacs of blood orange ‘Moro’ in vitro, which led the juices sacs showed a deeper red color (Fig. 5B and D). Moreover, in parallel with the increases in the anthocyanin content, the expression of *CitF3H* was significantly up-regulated by the blue light in the juice sacs of blood orange ‘Moro’ in *vitro* (Fig. 5C).

## Discussion

### ***CitF3H*** encoded a functional flavanone 3-hydroxylase in citrus fruit

To date, although *F3H* has been cloned and characterized in several plant species the research on *F3H* in citrus is limited and its roles in the regulation of flavonoid accumulation in citrus fruit are still far from being elucidated [33–36]. In this study, we identified a citrus flavanone 3-hydroxylase gene (*CitF3H*) using the sequence of *LcF3H* as a query in the *Citrus clementina* v.10 genome databases (http://www.phytozome.net/) and isolated it from three citrus varieties, Satsuma mandarin, Ponkan mandarin, and blood orange ‘Moro’. The amino acid sequence analysis showed that CitF3H had more than 80% identity with F3Hs reported in other plant species, which indicating that *F3H* was highly conserved in plants (Table S1). Moreover, the motifs for 2-oxoglutarate-dependent dioxygenase, and the amino acid residues that were responsible for polypeptide folding, ferrous iron ligating, and 2-oxoglutarate binding were strictly conserved in CitF3H of the three citrus varieties (Fig. 1). The further functional analysis confirmed that CitF3H catalyzed the formation of dihydrokaempferol from naringenin in vitro (Fig. 2). These results suggested that *CitF3H* isolated from the three citrus varieties encoded a functional flavanone 3-hydroxylase, which might be involved in the regulation of flavonoid biosynthesis in citrus.

### The expression of ***CitF3H*** was positively correlated with the anthocyanin accumulation in citrus juice sacs

In plants, *F3H* is a key structural gene in flavonoid biosynthetic pathway. It catalyzes the 3-hydroxylation of flavanone to form dihydrokaempferol, which is an intermediate for the biosynthesis of flavonols and anthocyanins. Previous studies suggested that *F3H* played an important role in the regulation of flavonols and anthocyanins accumulation in plants. In maize anthers, the expression of *F3H* was found to temporally coordinate with the appearance of flavonols [37]. Song et al. (2016) reported that overexpression of *LcF3H* in tobacco led to enhanced the accumulation of flavonols and flavan-3-ols [21]. In addition, *F3H* was also a rate-limiting enzyme in anthocyanin biosynthesis. The high expression of *F3H* led to the accumulation of anthocyanin in muscadine grapes and strawberry fruit [23, 38]. In this study, the results showed that the accumulation of anthocyanin was positively correlated with the expression of *CitF3H* in the juice sacs of the three citrus varieties. In blood orange ‘Moro’, the expression of *CitF3H* increased rapidly during the ripening process, which was well consistent with the accumulation of anthocyanin in the juice sacs. In Satsuma mandarin and Ponkan mandarin, in which anthocyanin was not accumulated, the expression of *CitF3H* kept constant at an extremely low level in the juice sacs during the ripening process. In citrus, flavonols are preferentially accumulated in leaves. In the fruit, however, a very small amount of flavonols is specifically accumulated in a few varieties, such as lemon and lime. In the present study, the analysis of flavonoid content and composition showed that flavonols were undetectable in the juice sacs of Satsuma mandarin, Ponkan mandarin, or blood orange ‘Moro’ during the ripening process. Although *CitF3H* was highly expressed, flavonols were not accumulated in the juice sacs of blood orange ‘Moro’, which indicating that *CitF3H* might be not the key gene controlling the flavonol accumulation in citrus fruit. Therefore, based on the results of flavonoid accumulation in the three different citrus varieties, it was suggested that *CitF3H* played crucial roles in the regulation of anthocyanin accumulation in the citrus juice sacs.

In this study, the functional analysis showed that *CitF3H* of Ponkan mandarin encoded a functional flavanone 3-hydroxylase; it catalyzed the formation of dihydrokaempferol from naringenin in vitro (Fig. 2). However, the expression of *CitF3H* in the juice sacs of Ponkan mandarin was extremely low and anthocyanin was not accumulated during the ripening process. Thus, these results indicated that the differential expression of *CitF3H* was a key molecular mechanism that regulated anthocyanin accumulation in the juice sacs of the three citrus varieties. In a previous study, the expression of *CitF3H* was investigated in *Citrus reticulate* and *Poncirus trifoliata* [34]. It was found that an indel variation in the promoter region of *CitF3H* led to different expression levels of *CitF3H* in the two citrus varieties. In the present study, to elucidate the different expression levels of *CitF3H* among Satsuma mandarin, Ponkan mandarin, and blood orange ‘Moro’, about 1000-bp promoter sequences of *CitF3H* were isolated and analyzed. The results showed that there was no genetic variation in the promoter of *CitF3H*, and promoter sequence of *CitF3H* shared 100% identity among the three citrus varieties. These results indicated that the differential expression of *CitF3H* might not be attributed to the promoter activity in Satsuma mandarin, Ponkan mandarin, and blood orange ‘Moro’.

In plants, it is well recognized that the regulation of anthocyanin biosynthesis at the transcriptional level is controlled by the MYB transcription factors. To date, two MYB transcriptional factors, *Ruby1* and *Ruby2*, have been identified in citrus [31, 39]. The citrus *Ruby1* and *Ruby2* can bind to the promoters of anthocyanin biosynthetic genes and activate the anthocyanin biosynthesis. In addition, *Ruby1* was specifically expressed in citrus fruit, which was controlled by the retrotransposon. In blood orange, an insertion of a Copia-like retrotransposon in 254-bp upstream of promoter activated the expression of *Ruby1*, and led to the accumulation of anthocyanin in the juice sacs. However, *Ruby1* was not expressed in most common citrus varieties because of lack of retrotransposon. In this study, we analyzed the *cis*-acting elements in the promoter of *CitF3H* by PLACE database, and found that promoter sequence of *CitF3H* contained three MYB bounding sites, which indicating that the expression of *CitF3H* might be regulated by the MYB transcription factors in citrus fruit (Fig. S2). To confirm it, we also investigated the expression of *Ruby1* in the juice sacs of the three citrus varieties in the present study (Fig. S3). The results showed that *Ruby1* was highly expressed in the juice sacs of blood orange ‘Moro’, while its expression level was extremely low in the juice sacs of Satsuma mandarin and Ponkan mandarin during the ripening process. The expression patterns of *Ruby1* were well consistent with those of *CitF3H*, which indicating that the differential expression of *CitF3H* might be regulated by *Ruby1* in the three citrus varieties.

### Blue light induced the expression of ***CitF3H*** and the accumulation of anthocyanin in the juice sacs of blood orange ‘Moro’

Light is one of the most important environmental factors influencing anthocyanin accumulation in plants. In citrus fruit, it was reported that bagging treatment (without light) inhibited anthocyanin accumulation in blood orange, while the biosynthesis of anthocyanins was restored when the bags were removed [40]. In the present study, the analysis of the *cis*-acting elements in the *CitF3H* promoter showed that eleven light responsive elements including five G-box elements were present in the up-stream1000-bp promoter sequence of *CitF3H*, which indicating that the expression of *CitF3H* was light inducible in citrus fruit (Fig. S2). The further experiments on the effects of light quality on the expression of *CitF3H* showed that the expression of *CitF3H* was induced by blue light, while it was not significantly affected by the red light in the juice sacs of blood orange ‘Moro’ in vitro. Under blue light, the increase in the expression of *CitF3H* was accompanied with the enhanced anthocyanins contents in the juice sacs of blood orange ‘Moro’ in vitro (Fig. 5). These results were consistent with previous studies, in which it was suggested that blue light was one of the most effective in inducing anthocyanin biosynthesis among the different light wavelengths.

## Conclusions

In this study, a *CitF3H* gene was isolated, and its molecular characterization was investigated in citrus fruit. The results showed that *CitF3H* was positively associated with the anthocyanin biosynthesis in the juice sacs of citrus fruit. In the juice sacs of two common citrus varieties of Satsuma mandarin and Ponkan mandarin, in which anthocyanin was not accumulated, the expression of *CitF3H* kept at an extremely low level during the ripening process. In contrast, *CitF3H* was highly expressed in the juice sacs of blood orange blood orange ‘Moro’. The high expression of *CitF3H* was well consistent with the accumulation of anthocyanin in juice sacs of blood orange ‘Moro’. In addition, blue light was an important factor for inducing the expression of *CitF3H* and accumulation of anthocyanin in the juice sacs of blood orange ‘Moro’. These results suggested that *CitF3H* was a key gene involved in anthocyanin biosynthesis in citrus fruit. The results presented in this study contributed to a better understanding of flavonoid biosynthesis in citrus, and provided new insights into improving the nutritional and commercial values of citrus fruit.

## Methods

### Plant materials

Satsuma mandarin ‘Miyagawa-wase’ (*Citrus unshiu* Marc.), Ponkan mandarin ‘Onta Ponkan’ (*C. reticulata* Blanco) and blood orange ‘Moro’ (*C. sinensis* Osbeck) grown at the Fujieda Farm of Shizuoka University (Shizuoka, Japan) were used as plant materials. Fruit samples were harvested periodically from October to February. Juice sacs were separated from the sampled fruit, and then immediately frozen in liquid nitrogen and kept at -80 ℃ until analysis.

To study the effects of blue and red lights on flavonoid accumulation, the juice sacs of blood orange ‘Moro’ (December) were excised and placed on 10 mL of MS medium supplemented 10% (w/v) sucrose and 1% (w/v) agar according to the method of Zhang et al. (2012) [41]. The juice sacs were irradiated with blue (470 nm, 100 µmol m^− 2^ s^− 1^) and red (660 nm, 100 µmol m^− 2^ s^− 1^) LED lights for 4 weeks at 10 ℃. The juice sacs cultured in the dark were used as the control.

### Functional analysis of CitF3H enzyme ***in vitro***

In this study, we conducted a Blast search in *Citrus clementina* v.10 genome databases (http://www.phytozome.net/) using the sequence of *LcF3H* (GenBank: KJ636468.1) as a query, which has been reported to encode a functional flavanone 3-hydroxylase in *Lycium chinese*. The cDNA of *CitF3H* isolated from Ponkan mandarin was cloned into the pCold GST vector (Takara Bio., Otsu, Japan). The recombinant plasmid was transformed into XL1-Blue *E. Coli* cells. After cultured overnight, 2 mL of culture of the transformants harboring the gene of *CitF3H* was inoculated in a 200 mL of 2×YT medium with carbenicillin (50 µg mL^− 1^). Cultures were grown at 37 ℃ until an optical density at 600 nm of 0.8 was reached. The culture solution was maintained at 15 ℃ for 30 min. The expression of proteins was induced by the addition of IPTG (final concentration, 1 mM), and the cultures were grown at 15 ℃ for additional 24 h. After centrifugation, the cells were harvested and frozen in liquid nitrogen, and then resuspended in extraction buffer (20 mM Na-Pi buffer pH 8.0, 10% glycerol). The suspensions containing the *E. coli* cells were lysed by sonication, and then 1% (v/v) Triton X-100 was added and shaken on ice for 30 min. After centrifugation at 5,000 × g for 90 min at 4 ℃, recombinant protein of CitF3H was subjected to TALON metal affinity resin immobilization (Takara Bio., Otsu, Japan). The His-GST-tagged proteins were then purified according to the manufacturer’s instructions, and imidazole was removed from the purified fraction using a PD-10 column (GE Healthcare, Chicago, IL, USA). The recombinant proteins were analyzed by SDS-PAGE with a 12.5% (w/v) polyacrylamide gel and WIDE-VIEW Prestained Protein Size Marker (Wako, Japan) using PhastSystem (Amersham Bioscience, US).

To investigate the enzymatic activity of CitF3H protein, the purified recombinant protein was reacted with flavanones (narirutin, naringenin, 3’-hydroxyflavanone, 4’-hydroxyflavanone, and hesperidin), flavones (apigenin, 4’,7-hydroxyflavone, 3’,4’-dihydroxyflavone, and 3’,4’,5,7-tetrahydroxyflavone), flavanols (dihydrokaempferol and myricetin). Reaction mixtures consisted of 100 mM Tris-HCl buffer (pH 7.2), 250 mM 2-Oxo-glutaric acid, 30 mM sodium ascorbate, 50 µM FeSO4, 10% (v/v) glycerol, 0.05% (v/v) Triton X-100, 500 µM substrates, and 40 µg of purified protein in a total volume of 100 µl. Assays were incubated at 37 ℃ with shaking for 2 h. The reaction solution was analyzed by HPLC and the new product was purified and analyzed by DART MS according to the method described by Seoka et al. (2020) [42].

### Extraction and determination of flavonoids and anthocyanin

The identification and quantification of flavonoids were conducted according to the method described by Seoka et al. (2020) [41]. Flavonoids were extracted from the freeze-dried juice sacs using a DMSO:methanol (1:1, v/v) solution. After homogenization, ultrasonication and centrifugation, the supernatant was filtered through a TORAST disc syringe filter (pore size 0.22 μm; SHIMADZU GLC Ltd., Japan). Then, the samples were analyzed using a HPLC system fitted with a YMC-UltraHT Pro C18 column (100 × 3.0 mm i.d. S-2 μm, 12 nm; YMC, Japan) at a flow rate of 0.6 mL min^− 1^. The eluent was monitored at 274, 310, 324, 338, and 362 nm using a MD4010 PDA detector. The flavonoid concentration was estimated by the standard curves and expressed as milligrams per gram dry weight. Flavonoid quantification was performed in three replicates.

Anthocyanins were extracted from the freeze-dried juice sacs using a HCl: methanol (1:99, v/v) solution. After homogenization, ultrasonication and centrifugation, the supernatant was filtered through a TORAST disc syringe filter (pore size 0.22 µm; SHIMADZU GLC Ltd., Japan). Then, the samples were analyzed using a HPLC system fitted with a YMC-UltraHT Pro C18 column (100 × 3.0 mm i.d. S-2 µm, 12 nm; YMC, Japan) at a flow rate of 1.0 mL min^− 1^. The eluent was monitored at 650 nm using a MD4010 PDA detector. A two-solvent gradient system of formic acid: milliQ water (1:9, v/v) (A) and formic acid: acetonitrile: methanol: milliQ water (4:9:9:16, v/v) (B) was used. The gradient program consisted four periods: (1) 0–35 min, 75%A, 25%B, (2) 35–45 min, 35%A, 65%B, (3) 45–50 min, 0%A, 100%B, (4) 40–69 min, 93%A, 7%B. The column temperature was operated at 30 ℃. In this study, two anthocyanin peaks were detected, and peak 1 was identified as cyanidin-3-glucoside according to the standard. Peak 2 was identified as cyanidin-3(6’’-malonyl) glucoside according to the MS analysis. The concentrations of all peaks were calculated with the standard curve of cyanidin-3-glucoside expressed as milligrams per gram dry weight. Anthocyanin quantification was performed in three replicates.

### RNA extraction and gene expression analysis

Total RNA was extracted from the juice sacs according to the method described by Ma et al. (2022) [43]. The RNeasy Mini Kit (Qiagen, Germany) was used to clean the extracted total RNA using on-column DNase digestion. The cDNA was synthesized with 2 µg of purified total RNA using a TaqMan Reverse Transcription Regents (Applied Biosystems, USA).

The gene expression was conducted by real-time quantitative PCR according to the method of Ma et al. (2022) [42]. As an endogenous control, the TaqMan Ribosomal RNA Control Reagents VIC Probe (Applied Biosystems) was used. The real-time PCR was performed using TaqMan Universal PCR Master Mix (Applied Biosystems) on a StepOnePlus™ system (Applied Biosystems). Each reaction contained template cDNA, 900 nM primers, and a 250 nM probe (Table S2). The thermal cycling conditions were 95 °C for 10 min, followed by 40 cycles at 95 °C for 15 s and 60 °C for 1 min. The data obtained from the StepOnePlus™ real-time PCR software (Applied Biosystems) was used to analyze the gene expression. The results were normalized with the results of 18 S ribosomal RNA. The Real-time quantitative RT-PCR was performed in three replicates for each sample.

### Statistical analysis

All values are shown as the mean ± SE for three replicates. The data were analyzed and Tukey’s HSD test (*P* < 0.05) was used to compare the different varieties or treatments.

## Electronic supplementary material

Below is the link to the electronic supplementary material.

Supplementary Material 1

## Data Availability

All data generated or analyzed during this study are included in this published article. *CitF3Hs* of Satsuma mandarin, Ponkan mandarin, and blood orange ‘Moro’ have been submitted to NCBI (https://www.ncbi.nlm.nih.gov/) with accession numbers: OQ148589- OQ148591.
