# Supplementary material for: Molecular characterization of a flavanone 3-hydroxylase gene from citrus fruit reveals its crucial roles in anthocyanin accumulation
Source: BMC Plant Biol. 2023 May 3;23:233. doi: 10.1186/s12870-023-04173-3 (PMC10155330; doi:10.1186/s12870-023-04173-3)
Supplement: Supplementary file 1 — Supplementary Material 1 [file 12870_2023_4173_MOESM1_ESM.docx]

**Molecular characterization of a flavanone 3-hydroxylase gene from citrus fruit reveals its crucial roles in anthocyanin accumulation**

Gang Ma^a,b^, Lancui Zhang^a^, Risa Yamamoto^b^, Nami Kojima^b^, Masaki Yahata^a,b^, Masaya Kato^a,b,^*

^a^ Department of Bioresource Sciences, Faculty of Agriculture, Shizuoka University, 836 Ohya, Suruga, Shizuoka 422-8529, Japan

^b^ Graduate School of Integrated Science and Technology, Shizuoka University, 836 Ohya, Suruga, Shizuoka 422-8529, Japan

*Corresponding author: Masaya Kato

Telephone: +81-54-238-4830 Fax: +81-54-238-4830

Email: kato.masaya@shizuoka.ac.jp

**Table S1.** Multiple sequence alignment of CitF3H with other plant F3Hs (%). The amino acid sequences of *Arabidopsis thaliana* (AtF3H, AAC49176.1), *Litchi chinensis* (LcF3H, ADO95201.1), *Dimocarpus longan* (DlF3H, ABO48521.1), *Canarium album* (CaF3H, AEO36935.1), *Theobroma cacao* (TcF3H, XP_007046698.1), *Gossypium barbadense* (GbF3H, KAB2051536.1), *Nekemias grossedentata* (NgF3H, AFN70721.1), *Vitis vinifera* (VvF3H, NP_001268034.1), *Paeonia suffruticosa* (PsF3H, AEN71544.1), *Malus domestica* (MdF3H, NP_001280854.1), *Eustoma grandiflorum* (EgF3H, BAD34459.1), Satsuma mandarin (OQ148589), Ponkan mandarin (OQ148590), and blood orange ‘Moro’ (OQ148591) are used multiple sequence analysis.

**Table S2.** Primers used for functional analysis and real-time PCR

| Gene |  | Sequences (5’→3’) |
| --- | --- | --- |
| *CitF3H* pCold GST | Forward | CTGGACATATGATGGCTCCTTCAACCCTCAC |
|  | Revserse | TATCCAGGTCGACCTAAGCCAAGATCTCCTC |
| *CitF3H* pGEX-6p-1 | Forward | TCGATGTCGACTCATGGCTCCTTCAACCCTCAC |
|  | Revserse | ATATAGCGGCCGCCTCTAAGCCAAGATCTCCTC |
| *CitF3H* | Forward | CGACCTCACTCTCGGACTCAA |
|  | Revserse | TTGGAGCAAGAGTGTGATGGTT |
|  | Probe | CGCCACACGGATC |
| *CitRuby* | Forward | GGGCAAGGGAATGACTTCAA |
|  | Revserse | GGTGGATTGTTTGCCGAGAT |
|  | Probe | ATTTGCAATTAGGAGATTAC |

**Figure S1.** The enzyme activity of pGEX-6P-1-CitF3H *in vivo*. The *E.coli* cells harboring the pGEX-6P-1-CitF3H construct was fed with naringenin, and HPLC analysis of the reaction product confirmed that dihydrokaempferol was produced as *in vitro* reactions. (A) HPLC analysis of naringenin. (B) HPLC analysis of new hydroxylated product of naringenin catalyzed by CitF3H. S: naringenin, P: naringenin reaction product.


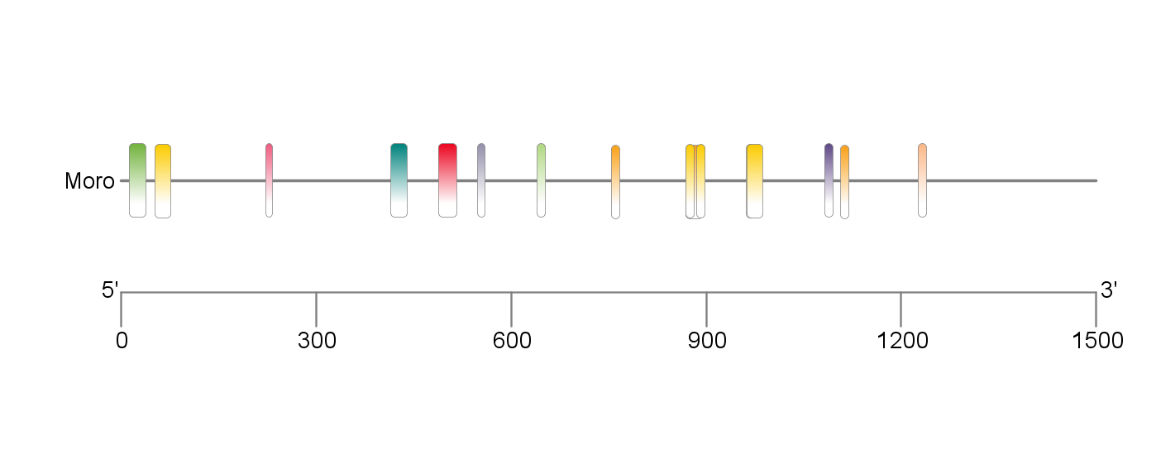

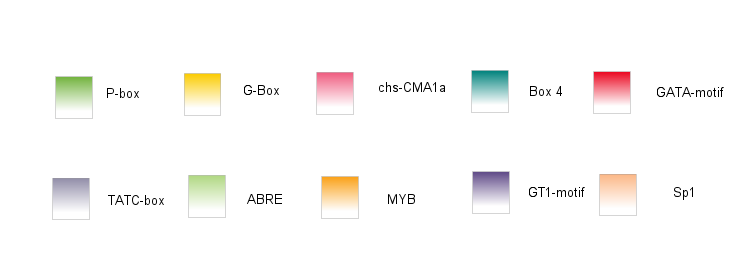


**Figure S2.** Analysis of *cis*-acting elements in the promoter of *CitF3H* by PlantCARE database (http://bioinformatics.psb.ugent.be/webtools/plantcare/html/). P-box, gibberellin-responsive element; G-Box, *cis*-acting regulatory element involved in light responsiveness; chs-CMA1a, light responsive element; Box 4, a conserved DNA module involved in light responsiveness; GATA-motif, a light responsive element; TATC-box, *cis*-acting element involved in gibberellin-responsiveness; ABRE, *cis*-acting regulatory element involved in light responsiveness; MYB, MYB binding site; GT1-motif, light responsive element; Sp1, light responsive element.

**Figure S3.** Gene expression analysis of *CitRuby1* in the juice sacs of Satsuma mandarin, Ponkan mandarin, and blood orange ‘Moro’ during the ripening process. The mRNA levels were analyzed by TaqMan real-time quantitative PCR. 18S ribosomal RNA was used to normalize the expression of gene in same conditions. Columns and bars represent the means ± SE (n=3), respectively. Different letters above each column indicate significant differences at *P* < 0.05 by Tukey’s HSD test.
